# Enhancing “theory of mind” through behavioral synchrony

**DOI:** 10.3389/fpsyg.2015.00870

**Published:** 2015-06-23

**Authors:** Adam Baimel, Rachel L. Severson, Andrew S. Baron, Susan A. J. Birch

**Affiliations:** Department of Psychology, University of British Columbia, Vancouver, BC, Canada

**Keywords:** theory of mind, social perspective taking, behavioral synchrony, ritual, interventions

## Abstract

Theory of mind refers to the abilities underlying the capacity to reason about one’s own and others’ mental states. This ability is critical for predicting and making sense of the actions of others, is essential for efficient communication, fosters social learning, and provides the foundation for empathic concern. Clearly, there is incredible value in fostering theory of mind. Unfortunately, despite being the focus of a wealth of research over the last 40 years relatively little is known about specific strategies for fostering social perspective taking abilities. We provide a discussion of the rationale for applying one specific strategy for fostering efficient theory of mind—that of engaging in “behavioral synchrony” (i.e., the act of keeping together in time with others). Culturally evolved collective rituals involving synchronous actions have long been held to act as social glue. Specifically, here we present how behavioral synchrony tunes our minds for reasoning about *other* minds in the process of fostering social coordination and cooperation, and propose that we can apply behavioral synchrony as a tool for enhancing theory of mind.

## Introduction

Philosophers have long debated the means by which we can, with any certainty, know of the mental worlds of others. This problem of *other* minds—that is *how* it is we think we know what other people know, feel and think—is not one that we can easily solve with logic alone (Dennett, 1981). However, throughout our evolution, humans have been endowed with the sufficient cognitive architecture that allows for us to, at the very least reason about the minds of others—our “theory of mind” (Premack and Woodruff, 1978; Wimmer and Perner, 1983; Baron-Cohen, 1999). This capacity for understanding others’ behaviors in terms of underlying mental states allows us to be empathic (Schnell et al., 2011), makes us adept cultural learners (Herrmann et al., 2007; Chudek and Henrich, 2011), and is involved in our moral reasoning (Moran et al., 2011; Young et al., 2011), our ability to coordinate and cooperate (Sally and Hill, 2006), as well as our ability to compete with, or manipulate, other individuals (Ybarra et al., 2007, 2010; Sher et al., 2014). Although this list is far from exhaustive, it should be clear that being an efficient mindreader facilitates successful navigation of the many challenges humans face in their socio-cultural environments. Indeed, those who are sometimes described as “mindblind”—individuals diagnosed along the autism spectrum—often experience tremendous hardships in everyday social interactions (Baron-Cohen et al., 1985).

Notably, being *able* to reason about other minds does not necessarily equate to being *accurate* at mindreading. Specifically, our reasoning about other minds is often inaccurate in one of two ways. For instance, when thinking about others’ minds could be most informative, such as when taking directions, we often fail to do so all together (Keysar et al., 2003; Samson and Apperly, 2010). Further, it is (extremely) common for individuals to think about and ascribe minds to entities when there is little to no evidence of a mind (at least not in the typical sense). For example, people frequently think about their computers as intentional beings with “minds of their own,” and people the world over ascribe mental states such as knowledge and intentions to bodiless spirits, ghosts, and gods (Waytz et al., 2010b). This set of inaccuracies represents systematic errors in mind perception and attribution—both true-misses and false positives. Moreover, our reasoning about the *content* of others’ minds is often inaccurate and systematically biased by our own perspectives and knowledge (Birch, 2005; Bernstein et al., 2011). This egocentrism prevents accurate attribution of, and subsequent reasoning about, the contents of other minds.

The mismatch between the human propensity for reasoning about other minds and our noted deficits in accurately doing so emerges from the imperfections of our evolved capacities, and the lengthy process of their development across the lifespan (Gehlbach, 2004; Brüne and Brüne-Cohrs, 2006). This gives rise to substantial individual variability in some domains of theory of mind such as emotion recognition and empathic tendencies (Baron-Cohen et al., 2001; Baron-Cohen and Wheelwright, 2004), while strikingly less so in others, such as reasoning about false beliefs (Liu et al., 2008). As such, what we refer to more generally as “theory of mind” is but a placeholder for a suite of related systems that function at different levels of cognitive processing. Implicit, automatic and inflexible systems for agency detection, face recognition, gaze following, emotion processing, joint attention, and our naïve theories of causality motivate a reflexive understanding of others’ behavior as resulting from underlying mental states (Apperly and Butterfill, 2009). This reflexive reasoning is elaborated with explicit, verbal and flexible thought (Epley and Caruso, 2008), only when we have the cognitive resources and motivation to do so (Rhodes, 2014).

As such, any account of how to foster theory of mind must take into consideration the various interconnected systems at play when people reason about the minds of others (Harwood and Farrar, 2010; Schaafsma et al., 2015). By understanding the parts of the process, we can begin to examine how to grease those gears and enhance our theory of mind capabilities. With such a framework in mind, we present behavioral synchrony, the act of keeping together in time with others, as a novel tool for honing and enhancing theory of mind. Specifically, we present evidence of the processes by which behavioral synchrony can correct for common inaccuracies in mental state reasoning by motivating directed reflexive mental state reasoning, and decreasing the egocentrism that would otherwise inhibit more explicit reasoning about others’ mental worlds.

## Music and Behavioral Synchrony

A recent study suggests that merely coordinating your actions with a complete stranger through participation in a musical game is sufficient to induce an empathic pain response of the same magnitude of that among very close friends (Martin et al., 2015). We argue that this choice of task, joint music making, is of special interest as it incorporates elements of synchronous action that are particularly capable of fostering theory of mind. The success of this intervention is particularly noteworthy considering the reported difficulties in enhancing theory of mind through explicit instruction. Specifically, studies that examine practicing and learning how to infer and engage with the minds of others in both typically developing and clinical samples (Ozonoff and Miller, 1995; Goldstein and Winner, 2012) remain inconclusive. Indeed, the difficulty in “teaching” theory of mind follows from the lack of a clearly defined relationship between experiential input (e.g., learning about mental states through parent–child discourse; Sabbagh and Callanan, 1998; Farrant et al., 2011) and cognitive scaffolding (e.g., executive function; Benson et al., 2013) in the ontogeny of a theory of mind. In contrast to explicit instruction, behavioral synchrony may offer unique opportunities to foster accurate mental state reasoning.

The production of music through coordinated rhythmic movement is a complex multimodal integration problem that humans are particularly capable of solving; we have got a knack for synchronizing our behavior with others and with signals in our environments (Overy and Molnar-Szakacs, 2009; Konvalinka et al., 2010). Establishing this synchrony, through spatiotemporal coordination to an external stimuli, is in and of itself a complicated dynamic task (Phillips-Silver et al., 2010). Yet, children within their first few years of life develop the ability to synchronize with others (Feldman, 2007; Kirschner and Tomasello, 2009). Early experiences of socially contingent, imitative, and synchronous behaviors help define the boundaries between self and other, while simultaneously allowing for effective navigation of those boundaries in fostering efficient interpersonal coordination (Nadel et al., 2005).

Across the lifespan, the ease with which we synchronize with others helps solve even the most mundane of joint coordination problems. Consider the complexity of the seemingly simple task of two separate minds and bodies figuring out how to lift and transport a heavy object. This requires those individual minds and bodies to perceive and react to each other, their respective movements and the constraints of the external world (Allport, 1924). Thus, sensory-motor coordination deficits can be particularly problematic in everyday life. Interestingly, movement abnormalities and deficits in spatiotemporal coordination are some of the earliest known precursors to diagnoses along the autism spectrum (Williams et al., 2001; Grossberg and Seidman, 2006) and are correlated to later deficits in empathic ability (Piek and Dyck, 2004). This connection between synchronous action and shared mental experiences—from keeping together in time, to keeping together in mind—is one that we are only recently beginning to understand.

Music and dance are the quintessential forms of coordinated human synchronous behavior. Ehrenreich (2006) and McNeill (1995) highlight the ubiquity of music, dance and drill in various forms of collective ritual throughout the anthropological and historical records while stressing the peculiar power behavioral synchrony has in both the management and rallying of large groups of physical bodies and, most strikingly, their mental states. In their now foundational studies, Wiltermuth and Heath (2009) empirically tested the core hypothesis laid out by Ehrenreich (2006) and McNeill (1995) that behavioral synchrony promotes cooperation. In a standard public goods game, groups that moved and/or sang synchronously, out-cooperated groups who did so asynchronously or did nothing at all by consistently contributing higher amounts to a shared account, from which all participants take an equal share. Synchrony fostered increased commitment to the group, and promoted greater feelings of liking, similarity and trust. Importantly, this heightened sense of group identity did not emerge when participants completed the very same task in an asynchronous manner—pointing to the effectiveness and specificity of behavioral synchrony in cultivating social cohesion above and beyond the effects of simply being part of a group. Indeed, such findings point to synchrony detection as one of the possible mechanisms responsible for establishing intergroup boundaries and for creating intergroup bias, speaking to its broad influence on social cognitive processing.

Attesting to the robustness of the effect of synchrony on cooperative behaviors, this result has since been replicated in more naturalistic settings (Cohen et al., 2010), and amongst diverse cultural groups (Cohen et al., 2013; Fischer et al., 2013). Further, the reported sensitivity to synchrony amongst conspecifics in promoting prosocial behaviors develops early (Kirschner and Tomasello, 2010) and emerges in infants as young as 14 months (Cirelli et al., 2014a). These converging lines of research provide strikingly clear evidence of how culturally evolved collective ritual practices around the world have galvanized this reliably developing cognitive connection between synchronous action and sociality.

However, there remains little consensus in this rapidly growing literature about the precise mechanisms by which these effects occur. Here, we put forth the hypotheses that in the process of fostering social cohesion and cooperation, behavioral synchrony enhances our capacities for theory of mind through two interrelated processes: (1) readying our minds for reflexive reasoning about mental states and (2) decreasing the egocentric biases that impede our accuracy in doing so. Of particular interest here, is not that synchrony fosters cooperation—but *how* it does so. Building from the literature on synchrony, cooperation and social cohesion, we lay the groundwork for understanding behavioral synchrony as a means to enhance theory of mind.

## Behavioral Synchrony and Reflexive Mental State Reasoning

Hove and Rise (2009) demonstrated that simply synchronizing with a visual target on a computer was not sufficient to induce increased affiliation with physically present others; it is really all about *interpersonal* synchrony. This connection between synchronizing with others and figuring out with whom to cooperate and affiliate seems to fall out of early developing inferences about our social worlds. In 14-month-old infants, for example, synchronous action functions as both motivator and cue for directing later, non-generalized, prosocial behaviors (Cirelli et al., 2014b). Kirschner and Tomasello (2010) have argued that in keeping in time with others, synchrony leads pre-school aged children to hold a representation of others in mind with a specific focus on the collective intention and shared attention that emerges from synchronous action. This capacity for sharing attention and intention emerges early in life and is a critical feature of the developing child’s theory of mind (Tomasello et al., 2005; Baillargeon et al., 2010).

In adults, synchronizing with others directs one’s attention toward those they have synched up with and in the process increases the likelihood with which they attribute them with personhood and mind (Macrae et al., 2008). Notably, synchrony induces greater memory for details of those with whom we synchronize with, but not greater generalized memory capacity (Miles et al., 2010). Thus, in the process of turning our attention toward those we synchronize with while increasing both the likelihood with which we attribute personhood to those individuals *and* hold this representation of the other in mind, behavioral synchrony engages the cognitive systems that ready our minds for thinking about the mental states of others.

Furthermore, synching up with others makes us better able to infer and predict other’s future behaviors, increasing not only cooperative tendencies, but also the *ability* to successfully cooperate. In one study (Valdesolo et al., 2010), participant dyads were instructed to either rock in or out of synchrony with each other in chairs, and then worked together in navigating a steel ball through a wooden labyrinth. Success on this task was determined by the ease with which participants could infer and predict their partners’ subtle movements, while dynamically adjusting their own, without the use of verbal communication, in order to quickly get the ball through the maze. Synchronous pairs, compared to asynchronous pairs, were significantly quicker at navigating the ball through the labyrinth. Further, success on this task was mediated by a synchrony-induced increase in the ability to detect subtle differences in temporal movement on an unrelated task. That is, participants were better able to accurately report whether a ball on a screen moved at the same or a different pace (which varied across trials) after passing behind an opaque rectangle; raising the interesting possibility that moving in synchrony with others promotes a domain-general increase in ability for tracking agency—another early developing feature of our core cognitive capacity for theory of mind (Gergely et al., 1995; Baron-Cohen, 1999; Johnson, 2000).

Collectively, these lines of research provide convergent evidence for the various ways in which behavioral synchrony prepares us for engaging with the mental world of others’. By fostering shared and other-directed attention, individuals in synchrony become acutely aware of what others’ perceive, making the jump from what others see to what others think cognitively easier to compute (Sebanz et al., 2006). Further, the act of synchronizing, keeping to the beat, in and of itself dictates not only what others should do, but will do. Thus freeing up cognitive resources otherwise spent on predicting others’ behaviors, allowing for, as will be described below, more explicit reasoning about others’ mental worlds.

## Behavioral Synchrony, Egocentrism, and Psychological Distance

In creating a sense of “we” amongst previously unrelated individuals, behavioral synchrony has been consistently demonstrated to foster increased liking, feelings of similarity, and affiliation (Haidt et al., 2008; Hove and Rise, 2009; Wiltermuth and Heath, 2009; Lakens and Stel, 2011; Valdesolo and DeSteno, 2011). Synchrony actually makes us less able to distinguish our own faces from those of whom we have synched up with (Paladino et al., 2010)—blurring the boundaries between self and other. In this act of getting over one’s self, behavioral synchrony may engage and foster explicit mental state reasoning through a reduction of our egocentric biases that otherwise hinder our ability to reason about another’s perspective.

Further, psychological distance can inhibit the social cognitive processes involved in mental state reasoning. The larger the psychological distance between two individuals or entities (e.g., the greater the dissimilarity), the less likely they would believe they shared any meaningful connections, attitudes, traits, and of particular interest here, the less likely they would be to attribute minds to each other (Waytz et al., 2010a). When asked to think about others who are perceived as psychologically distant (e.g., the homeless), individuals dehumanize others and fail to even recruit the brain networks used in everyday social cognitive processes (Harris and Fiske, 2006). Further, simply tagging others as not being in-group members has been demonstrated to be sufficient for upping the threshold of mind perception—requiring more humanness (on a doll and human face-morphing task) before we willingly attribute them with mental states (Hackel et al., 2014). Interestingly, naturally occurring synchrony in dyadic interactions occurs significantly less when interacting with psychologically distant others (Miles et al., 2009). Synchrony then, when experimentally induced in the lab or experienced through collective ritual, might aid in decreasing psychological distance (Vacharkulksemsuk and Fredrickson, 2012), and increase the likelihood with which we explicitly engage with and reason about others’ mental worlds.

## General Discussion and Future Directions

The act of keeping together in time with others, participation in synchronous collective ritual, binds individuals into cohesive groups (McNeill, 1995; Ehrenreich, 2006). This is but one culturally evolved solution to the problem of sustaining large-scale cooperation in groups (Henrich and Henrich, 2007). Here, we argue that synchrony fosters cooperation by exploiting our everyday social cognitive reasoning about other minds. That is, by directing attention to others and their mental states, while decreasing the perceived psychological distance between individuals, behavioral synchrony makes us better able to reason about other minds and thus coordinate and cooperate. Behavioral synchrony then, like the human propensity for imitation, is part of a larger suite of processes that allow for effective interpersonal coordination between physical bodies *and* minds (Chartrand and Lakin, 2013). The human capacity for interpersonal coordination and cooperation is remarkable, and known to reliably recruit neurological systems involved in mental state reasoning (McCabe et al., 2001; Balslev et al., 2006; Lissek et al., 2008). In turn, the ease with which one reasons about others’ mental states has been linked to the ability to successfully coordinate in joint-action paradigms (Humphreys and Bedford, 2011; Curry and Chesters, 2012).

The connection between behavioral synchrony and the cognitive systems we use to engage with others’ mental worlds underscores the interconnectedness of our behavior- and mind-reading abilities. Theory of mind is not telepathy—it is a complex inferential and predictive process that attempts to make sense of real cues that exist out there in our environments—behaviors (Whiten, 1996). Following a simulationist perspective on the mechanisms underlying theory of mind processes (Gallese, 1998; Frith and Frith, 2006), when we move together in time with others, understanding their behavior becomes a much simpler task as the behavior of others is matched in our own, making it cognitively less demanding and less difficult to reason about their mental states (Keller et al., 2014).

Research explicitly exploring how behavioral synchrony can foster theory of mind is sparse. However, there is an emerging literature examining the benefits of musical group interaction and dance-movement therapy in enhancing various components of theory of mind in typically developing and clinical samples of young children. Both of these applications heavily involve synchronous interactions with others and are found to increase social cognitive processes that promote reflexive mental state reasoning (e.g., joint attention, imitation, gaze following; Landa et al., 2011), as well as explicit tendencies for empathy and perspective taking (McGarry, 2011; Behrends et al., 2012; Rabinowitch et al., 2013).

Thus, the question remains as to how broadly the current hypotheses can be put into practice. We are currently building an in-depth research program exploring how behavioral synchrony can differentially enhance different components of theory of mind—directly testing the hypotheses that synchrony fosters both explicit and implicit mind perception, empathy and perspective taking using a battery of measures built specifically to capture correlates of theory of mind. With promising results, the question of what this synchrony-induced mental state reasoning accomplishes in terms of other processes we know to be related to theory of mind—such as in the domains of moral reasoning and cultural learning—remains open for future research. Our hope is that we have made a case here for the viability of this research program and inspire future research toward the goal of better understanding the mechanisms underlying mental state reasoning in order to foster better social perspective taking.

What emerges from an understanding of the connection between behavioral synchrony and theory of mind is a cohesive framework from which to understand the already well-established effects of synchrony on coordination, cooperation and cohesion—understanding the process by which joint physical action leads to joint mental connection. This framework provides answers to (or at the very least testable hypotheses regarding) the question of why behavioral synchrony is so ubiquitous in collective rituals around the world. From army drills to church choirs, culturally evolved collective rituals involving synchrony tune our minds for reasoning about other’s mental states. In doing so, individuals become better able to learn from, coordinate, cooperate, and empathize with others—shaping human sociality. Presently, we argue that we can exploit this culturally galvanized connection between synchrony and mental state reasoning—and apply synchrony as a tool for fostering theory of mind.

### Conflict of Interest Statement

The authors declare that the research was conducted in the absence of any commercial or financial relationships that could be construed as a potential conflict of interest.
